# Machine Learning Methods for Analysis of Metabolic Data and Metabolic Pathway Modeling

**DOI:** 10.3390/metabo8010004

**Published:** 2018-01-11

**Authors:** Miroslava Cuperlovic-Culf

**Affiliations:** Digital Technologies Research Center, National Research Council of Canada, 1200 Montreal Road, Ottawa, ON K1A 0R6, Canada; cuperlovim@nrc.ca; Tel.: +1-613-993-0116

**Keywords:** system biology, metabolomics, metabolism modeling, machine learning, genomics

## Abstract

Machine learning uses experimental data to optimize clustering or classification of samples or features, or to develop, augment or verify models that can be used to predict behavior or properties of systems. It is expected that machine learning will help provide actionable knowledge from a variety of big data including metabolomics data, as well as results of metabolism models. A variety of machine learning methods has been applied in bioinformatics and metabolism analyses including self-organizing maps, support vector machines, the kernel machine, Bayesian networks or fuzzy logic. To a lesser extent, machine learning has also been utilized to take advantage of the increasing availability of genomics and metabolomics data for the optimization of metabolic network models and their analysis. In this context, machine learning has aided the development of metabolic networks, the calculation of parameters for stoichiometric and kinetic models, as well as the analysis of major features in the model for the optimal application of bioreactors. Examples of this very interesting, albeit highly complex, application of machine learning for metabolism modeling will be the primary focus of this review presenting several different types of applications for model optimization, parameter determination or system analysis using models, as well as the utilization of several different types of machine learning technologies.

## 1. Introduction

Metabolism is the basic process in every biological system providing energy, building blocks for cell’s development and adaptation, as well as regulators of a variety of biological processes. It is becoming generally understood and accepted that metabolomics delivers the closest insight into physiological processes, providing valuable data for disease diagnostics, toxicological studies and treatment follow-up or optimization in a variety of applications from clinical to environmental or agricultural sciences. Recent analysis has shown that in the domain of precision medicine metabolomics provides highly complementary information to next generation sequencing, allowing a very good method for distinguishing between genes that are actually causing disease or are benign mutations, therefore helping in disease risk assessment and customization of drug therapy [1]. In addition, accurate modeling of metabolism, made possible with improved computer technology and the availability of a large amount of biological data, is becoming increasingly important for a number of highly diverse areas from bioreactor growth, drug target determination and optimization and testing to environmental bioremediation, for example. Many of these applications produce large amounts of data requiring different aspects of analysis and allowing derivation of different knowledge including sample or biomolecule clustering or classification, the selection of major features and components, as well as the optimization of model parameters. Machine learning has been used for all of these tasks. 

Machine learning has been defined in many ways, from “a field of study that gives computers the ability to learn without being explicitly programmed” [2] to the more specific and descriptive definition given by Mechell [3]: “a computer programs is said to learn from experience (E) with respect to some class of tasks (T) and performance measure (P) if its performance at tasks in T as measured by P improves with experience E” (Box 1). Although machine learning is an old area of computer sciences, the availability of sufficiently large datasets providing learning material, as well as computer power providing learning ability resulted in machine learning applications flourishing in biological applications only recently. Machine learning methods can be grouped by algorithm similarities as shown in Table 1. Many of these methods have been used in metabolomics analysis, and some related references are provided in the table. Several methods have been used for cell metabolic network modeling, and those will be discussed in greater detail below.

Box 1 **Definition of several basic terms used in machine learning and throughput this text.**

**Unsupervised learning** algorithms identify patterns from data without any user input providing for example data-driven sample separation or feature grouping based only on the data;

**Supervised learning** algorithms use previously labeled data (training set) to learn how to classify new, testing data. This approach determines major features or combinations of features that can provide the highest sample label accuracy in the training set and uses this information for future input labelling.

**Classification** is a process of mapping input data to a discrete output, e.g., sample label. The quality of classification results is determined using accuracy, i.e., sensitivity and specificity;

**Regression** is a process of mapping input data to a continuous output variable, e.g., quantity or the size. The quality of regression prediction is determined using root mean squared error.

**Figure 1:**
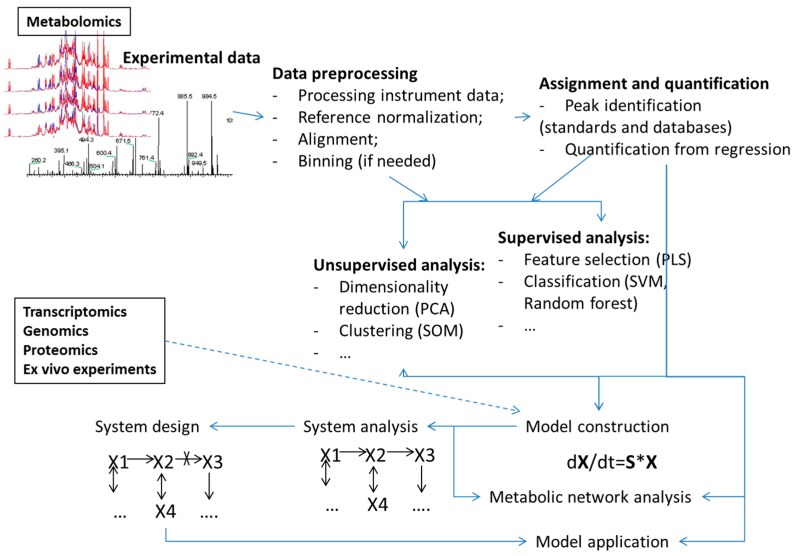
Overview of different data analysis steps in metabolomics and metabolism modeling where machine learning methodologies have found uses.

Machine learning methods have been used for analyses in all steps of the metabolomics process (Figure 1) including:

- Analyses of points or bins from the metabolomics measurements such as NMR spectra or MS spectrograms (examples of use of PCA and PLS methods are too many to reference). A recent example of an application of deep learning, as well as SVM, RF and several other machine learning algorithms was presented by Alakwaa et al. [20];
- Assignment of peaks in spectrograms or spectra for metabolite identification [12,21];
- Quantification of metabolite concentrations from high throughput data [22,23];
- Selection of a sets of the most informative attributes (“feature selection”) for given sample groups [19,24];
- The combination of features or points using certain rules (e.g., principal components) aimed at reducing the number of features for analysis or selection of major variances (reviewed in, for example, [25,26]).

Examples include unsupervised and supervised [5] type analysis either trying to group samples or features without any user input or bias or trying to get the best classification models based on user-labelled training data, respectively. Several reviews provide a general overview of the application of machine learning in omics [27,28] and metabolomics [29,30,31,32], as well as clinical application of metabolomics through machine learning [33,34]. Analyses have utilized several commercial tools, as well as many freely available applications with some highly useful, freely available software providing straight forward application of machine learning listed in Table 2. Other metabolomics software, some including machine learning methods, are listed in [35,36], in earlier publications [37,38] and also within the R package. In addition, the number of freely available tools provides ways for an all-inclusive workflow in metabolomics analysis from data preprocessing all the way to metabolic network analysis. Examples of such tools are mummychog [39], Cytoscape [40] and Galaxy [41].

Several different applications of machine learning in modeling of cell metabolism will be presented in an attempt to show the variety of possibilities that can result from combining metabolism modelling, omics data and machine learning rather than trying to show all examples of machine learning utilization in metabolism modelling and metabolomics.

## 2. Metabolism Modeling

Integrated analysis of the heterogeneous, high-throughput omics data is necessary in order to determine true factors leading to observed or possible physiological states, to obtain the most sensitive and specific markers or fully understand the effects of treatments, pathogens or toxins. Mathematical and computational models provide platforms for the integration of disperse knowledge and for testing and prediction of behaviours under different environments or following different manipulations. In other words, *in silico* models provide an avenue for a controlled analysis of targets, biomarkers or cell growth under different conditions. In this way, modeling can help unravel complex diseases while at the same time allowing the determination of possible treatment targets or in silico testing of drug or toxin effects. 

**Figure 2:**
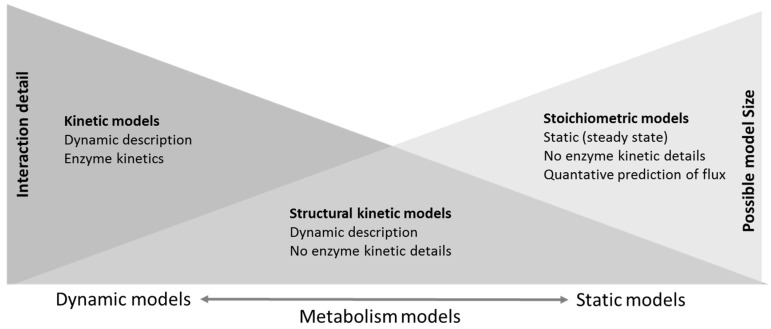
Schematic comparison of representative methods for the metabolic pathway and network models including different constraints and approaches to defining metabolic reactions.

Any biological process, including metabolic processes, can be represented as a pathway with interconnected nodes, and thus, mathematical models such as graph theory, ordinary differential equations, Petri nets, etc., have an obvious role in the simulation of biological systems. For a structural view, a metabolic network can be perceived as a bipartite graph that consists of two sets of nodes representing metabolites and biochemical processes. Graph visualization of the metabolic network provides a way to gather and combine information from the literature, expert knowledge, public databases, as well as omics results, but does not allow simulation of the processes in the network. In a simulation, the dynamics of chemical processes in the metabolic network can be described at various levels of detail. Models can, on one side, include comprehensive kinetic representation of each included pathway thus allowing accurate prediction of the dynamic behavior of a subset of pathways or, at the other extreme, the genome scale models of the whole network can be subjected to stoichiometric analysis with constrained parameter ranges and the assumption of the steady state while allowing modeling of a complete cell network showing the steady state behavior of cells or even organisms (Figure 2). Between these two extremes are for example structural kinetic models [47], the analysis of “superpathways” [48] or models using Petri nets initially proposed by Reddy et al. [49] and extensively reviewed by [50,51], providing some level of dynamic analysis of larger, albeit not complete, networks. In all cases, models are based on prior knowledge. A very interesting and detailed classification of metabolism modeling methods has been presented by [52], and a greater level of detail is shown in the associated mind map [52].

Detailed kinetic models have been built from ex vivo enzymatic measurements and Michaelis–Menten or related kinetic laws with the possibility for optimization of a few factors using experimental data and optimization methods. An increasing availability of genomics data and a variety of highly innovative bioinformatics techniques led to an ever-increasing number of biological networks constructed and presented [53]. Genome-scale metabolic networks allow constraint-based flux analysis in stoichiometrically-defined networks, ultimately encompassing all known processes in organisms with the possibility for adaptation to the metabolism of specific cells, as well as modeling of groups of cells present in an organ [54]. However, the inference of biological network parameters from experimental data remains a major challenge due to the dynamic nature of the system, the non-linearity of the systems and the fact that only a fraction of components and environments in the system can be measured. Incomplete knowledge of many feedback and feed-forward controls [55] and the incompleteness of detailed knowledge concerning the effects of inhibitors, gene mutations and environments, on one side, with the increasing availability of different types of omics data describing a variety of cellular systems (e.g., genomics, metabolomics measured in different tissues, longitudinal studies or following different treatments) makes machine learning application to this problem highly desirable [56]. This is further compounded by the importance of biological questions that can be resolved using accurate, targeted metabolic network models and the relational spatial and temporal structure of interactions between the involved molecules [56]. 

## 3. Machine Learning in Metabolism Modeling

Machine learning algorithms have been used to build or optimize kinetic and genome-scale models from example data in order to make data-driven predictions or conclusions [53,57,58]. For existing models, machine learning algorithms have been used to determine the essentiality of features in a network (reviewed in [57]). Furthermore, machine learning, for example clustering or SVM, can be used to map genomics, proteomics or metabolomics data onto a metabolic model where different omics and model data can be integrated in multi-view machine learning algorithms with possibly different machine learning algorithms analyzing each omics layer followed by the aggregation of layers as shown with the methods developed in multi-layer network theory [52].

Regardless of the application, the development of predictive models using a machine learning approach is accomplished in several steps: (a) selection of learning attributes; (b) construction of training and test sets; (c) selection of learning algorithms; (d) design of the machine learning approach; (e) evaluation of the predictive performance of models. All of these steps have to be considered and optimized in machine learning development or the application of metabolism models, either kinetic, genome scale or mixed. In the following, we will present examples of machine learning application in kinetic, genome-scale and mixed model development and their applications.

**Figure 3:**
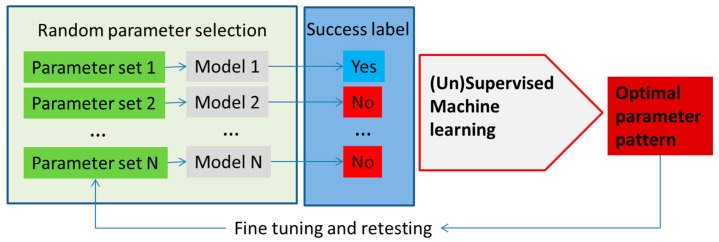
An outline of the utilization of machine learning methods in metabolism modeling application for optimization of parameters in the model, as well as testing different input conditions. In this example, parameters in the model are selected at random (e.g., using Monte Carlo sampling) or, alternatively, reactions are taken out or put in (in the constraint-based models) and the models are run. The success of each model is determined using the parameter of interest (e.g., cell growth or cell death, production of the molecule of interest, etc.). Model parameters (1–N) are used as feature vectors with the success label used as the class label in the machine learning classifier. The classifier determines patterns in the parameter space with the highest discriminatory power that ensures success according to the model.

Machine learning methods can enhance the development and application of metabolism models with applications broadly divided into model parameter determination, metabolic network analysis and model application. A schematic, highly simplified example of the model parameter determination is outlined in Figure 3. 

Parameters used in metabolic models, kinetics or flux ranges can be determined using either the in vitro enzymatic assay in the bottom-up approach or can be indirectly inferred from metabolomics time series data in the top-down method. The bottom-up approach for the development of kinetic models requires experimental measurements of the kinetics of each enzyme providing information that is then integrated in the model. This approach is highly experimentally demanding, and although information has been made available for many enzymes leading to a number of kinetic models of some major pathways, the bottom-up approach is unlikely to provide information for all enzymes, particularly when considering possible kinetic changes under different conditions present in vivo (e.g., inhibition, protein interactions). Optimization of kinetic parameters can be done from metabolomics time series data. The majority of methods use optimization algorithms that try to find the best model parameters from available experimental data. Examples of the utilized optimization methods are: genetic algorithms, evolutionary programming, simulated annealing, Newton–Raphson and Levenberg–Marquardt methods (reviewed in [59]). However, detailed, thermodynamically-minded kinetic models of metabolism include complex, often non-linear relations between metabolites, as well as heterogeneous parameters (e.g., kinetic parameters, concentrations), making the utilization of fitting strategies difficult. 

Saa and Nielsen [58,60] have proposed the application of Approximate Bayesian Computation (ABC) in order to determine the joint parameter, posterior, distribution that can explain the experimental data. The ABC approach determines whether the distance between the experimental and simulated dataset is below a predetermined threshold in order to decide whether to accept the parameter vector used to generate the simulated dataset. The kinetic parameter calculation using this method relies on the Monod–Wyman–Changeux model and assumes independence between the rate of reaction catalyzed by the enzyme and the reactions regulating the enzyme’s activation, leading to a rate of reaction that can be calculated as a product of the catalytic rate, and the enzyme activation rate General Reaction Assembly and Sampling Platform (GRASP) has been developed to determine the distribution of allowed kinetic parameters from available data. The detailed kinetic model for each enzyme in the network is built by fitting experimental data to simulations using the ABC-sampling method. ABC greatly increases the range of kinetics covered when compared to the heuristic Ensemble Modeling (EM) approach for kinetic model construction and parameter fitting. Using this approach, the authors have developed and presented a detailed kinetic model for the methionine cycle. 

Decision trees have been used to search for discriminating patterns in the model parameter space in structural kinetic modeling [47,61,62], but other machine learning methods could be explored as well. Structural kinetic models do not require explicit knowledge of the rate equation, but instead try to develop parametric representation of the Jacobian of the system [47,63]. In an example, the optimal parameter pattern is determined from the large number of structural kinetic models based on randomly sampled parameter sets. Supervised machine learning, in the published, case decision trees, was used to determine the patterns of parameters that lead to the highest discrimination between classes of simulation results based on user predefined criteria (Figure 3). Using this approach, the authors have developed a detailed structural kinetic model of the Calvin–Benson cycle and connected pathways that could then be used for the analysis of the effect of regulators [61,62]. Structural kinetic modeling can be performed using the MATLAB platform presented by Girbig et al. [61] where this tool can provide simulations of models with different patterns (using Monte Carlo sampling) that can then be examined using machine learning methods.

**Figure 4:**
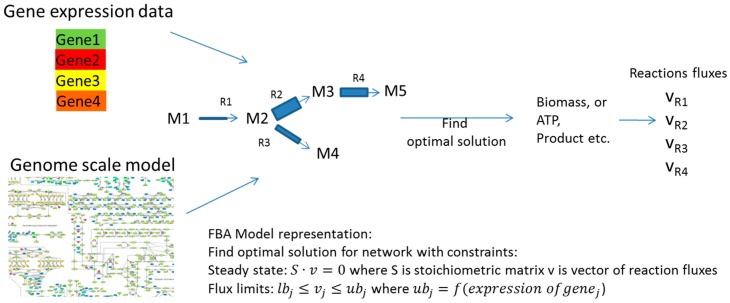
In the examples presented by [71,72], gene expression data are used to define the upper limit for fluxes across reactions catalyzed by that gene. Further flux optimization is subsequently possible with different methods including machine learning, as shown in Figure 3.

**Figure 5:**
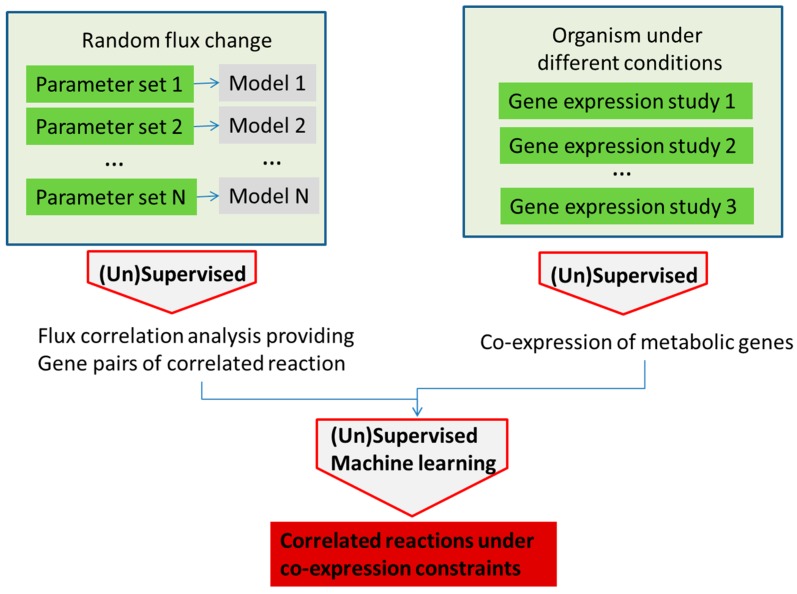
Analysis of gene correlation in stoichiometric models and experimental gene expression data can provide information about the system robustness and lead to more detailed information about gene correlation under different conditions.

Both kinetic and structural kinetic models provide very detailed simulation avenues for the subset of pathways. Metabolic network models, on the other hand, try to describe all reactions that are available to an organism with every node in the network representing an intermediate in a chain of chemical, metabolic reactions. These large networks can be modeled in the constraint-based approach where models have an associated solutions space in which all feasible phenotypic states exist under imposed constraints. Typically in these models, metabolite flow is constrained by network topology, the steady state assumption and by upper and lower bounds for each individual reaction flux. The challenge then in this type of modelling is to determine and impose major constraints in order to define and investigate the solution space in a way that determines physiologically-relevant fluxes or phenotypes [64,65,66,67,68]. Constrained network-scale model development and application can be done using the very popular COBRA platform (COnstraint-Based Reconstruction and Analysis Toolbox) [69], as well as related tools such as MONGOOS (MetabOlic Network GrOwth Optimization Solved Exactly) [70]. In one approach, flux limits can be obtained from gene expression data as shown by [71,72] (Figure 4). Machine learning can be used for model optimization or the determination of major fluxes once again using the general approach shown in Figure 3. An interesting example of the use of machine learning for the improvement of models by comparing the empirical interaction data and model predictions was presented by Szappanos et al. [73]. In this example, machine learning, in this case, the random forest method, was used to determine whether modifying the constraint-based model can increase its predictive power. Similarly, the effect of removing reactions, modifying reaction reversibility and altering the biomass function in the model was assessed against empirical data for the effect of several inhibitors on gene expression in *Mycobacterium tuberculosis*, and this type of analysis was used to determine targets for inhibitors, as well as suggesting several possible modulators of the production of mycolic acid by this bacterium [71] (Figure 5). 

Genome-scale stoichiometric models lack information about enzyme kinetics and cannot accurately account for the response of metabolism to changes in enzyme expression or enzyme inhibition. Development of large, genome-scale kinetic models is therefore of great interest; however, it is still hampered by uncertainty in metabolite concentration levels and thermodynamic displacement, as well as uncertainty in the kinetic properties of enzymes [63,74]. Towards the determination of accurate ranges of kinetic parameters for genome-scale level models with kinetic consideration, Andreozzi et al. [74] have utilized machine learning methods in combination with kinetic modeling principles in the approach named iSCHRUNK. In this approach, machine learning uses values of observed data samples to infer parameter ranges that predict whether data can satisfy a given property. The goal of the method is to determine ranges for a set of parameters: *p*_1_, *p*_2_, …, *p*_n_ so that function *f*(*p*_1_, *p*_2_, …, *p*_n_) satisfies the given property. In the presented case, the requirement was that *f*(*p*_1_, *p*_2_, …, *p*_n_) <0 without knowing the exact functional form of *f*(*p*_1_, *p*_2_, …, *p*_n_). With an adequate training dataset, machine learning, in this case, the decision tree learning algorithm, was able to determine allowed ranges for kinetic parameters of metabolic reactions that were consistent with observed physiology. The ORACLE approach combined with machine learning iSCHRUNK analysis of allowed ranges for kinetic parameter provides a very interesting approach for the creation of genome-scale kinetic models that will be increasingly feasible and increasingly accurate as more data become available.

Metabolic pathways have to be sufficiently robust to be able to tolerate fluctuations in protein expression levels, as well as changes in environmental conditions. Analysis of metabolic models can thus help in identifying genes that might be essential for cell growth, survival or production. Machine learning models can be trained to predict and classify genes of an organism as essential or non-essential based on a training set of known, labelled essential or non-essential genes. A number of different machine learning methods has been used to try and determine gene essentiality from metabolic network information, including: SVM, ensemble-based learning, probabilistic Bayesian methods, logistic regression and decision tree-based methods (reviewed in [75]). Plaimas et al. [76] have presented a machine learning strategy for the determination of essential enzymes in a metabolic network aiming towards the determination of interesting drug targets. Ensemble modeling has been investigated as an approach for robustness analysis [77]. Ensemble learning is the approach of creating a high-quality ensemble predictor by mixing results from many weak learners. Ensemble learning methods have been particularly useful for the prediction of essential genes and proteins from network data. An example of the ensemble learning method is random forests, which combine two machine learning techniques: bagging and random feature subset selection for predictions. Nandi et al. [75] present a very interesting approach for the selection of essential features using the SVM-RFE machine learning approach. In their example, the authors used a genome-scale metabolic network of *Escherichia coli* to create reaction-gene combinations and label the essentiality of each combination based on experimental data. The authors also combined the data from Flux Coupling Analysis (FCA) in order to account for the inherent limitations of metabolic network flux distribution analysis on environmental dependences. FCA was included as one of 64 features describing each reaction-gene combination. SVM-RFE was trained using experimentally-determined gene essentiality information and 64 features determined for each reaction-gene combination providing information about major features and essential genes.

A method, SELDOM (enSemble of Dynamic lOgic-based Models), was recently presented by Henriques et al. [78], attempting to help determine dynamic network models from experimental data. Ensemble learning methods try to improve predictive performance by utilizing multiple learning algorithms to create a set of classifiers that are all used to determine the best classification. In the case of SELDOM, ensample learning is used to build a set of dynamic models from experimental training data. Individual predictions of these models are combined, and spurious interactions in the combined model are removed in order to reduce the danger of overfitting. This is a fully data-driven approach that does not require any prior knowledge about either the network or the dynamics of reactions. This method has been developed and tested for modelling signaling pathways, but a similar methodology can be applied for modeling of the metabolic network from data. Uniquely, SELDOM develops models of the network dynamics using ordinary differential equations, and once the model is created, it can be used to interpret or predict networks’ behavior in new experimental conditions. This approach of course depends on the availability of appropriate, time course measurements.

Metabolomics or genomics data can be used to discover unknown metabolic pathways and their regulation, as well as to determine preferred metabolic routes in a particular system with a known metabolic network. In this application, the goal is the construction of correlation and causation networks between metabolites. The correlation network determines the probable relation between metabolites and enzymes using usually statistical analysis of measurements from different biological conditions. The correlation network provides static information about the most likely network. The causation network is usually obtained from time series metabolomics data and provides cause-effect information about the metabolites in the network. Some examples of methods used for causation network determination are dynamic Bayesian models, Granger causality approaches, as well as the BST-loglem approach, which combines mathematical and statistical methods [63].

Machine learning has been used to predict inhibitory effects of substances on the metabolic network. In the examples presented by Tamaddoni-Nezhad et al. [79,80], background knowledge including network topology and functional classes of inhibitors and enzymes, although incomplete, was used to interpret NMR metabolomics measurements of urine samples following injection of toxins. In that work, logic-based representation and a combination of abduction and induction methods was used to model inhibition in the metabolic network aimed towards prediction of inhibitory side effects of drugs. Abductive learning encompasses methods for finding the best explanation of observations. In the work of Tamaddoni-Nezhad et al., the abduction approach was utilized to form hypotheses related to enzyme inhibition by drugs from the NMR metabolomics data. In inductive learning, the method tries to determine a general rule from observations resulting in assignment of a class label to the input. In the example presented in [79,80], inductive learning was used to generate knowledge that can come either directly or indirectly through the current theory, but can extend and add new interrelationships and new links between relations in the model. The abduction and induction learning can be subsequently performed in a cycle continually adding explanations of the observations to the theory, i.e., model. This approach has been deployed in Inductive Logic Programming (ILP) with specific application for metabolic network analysis presented in Progol 5.0 with a detailed example of its use presented in [56,79]. Lodhi and Muggleton [1] have also presented the application of stochastic logic learning (SPL) in ensemble learning for the estimation of rates of enzymatic reactions in metabolic pathways.

**Figure 6:**
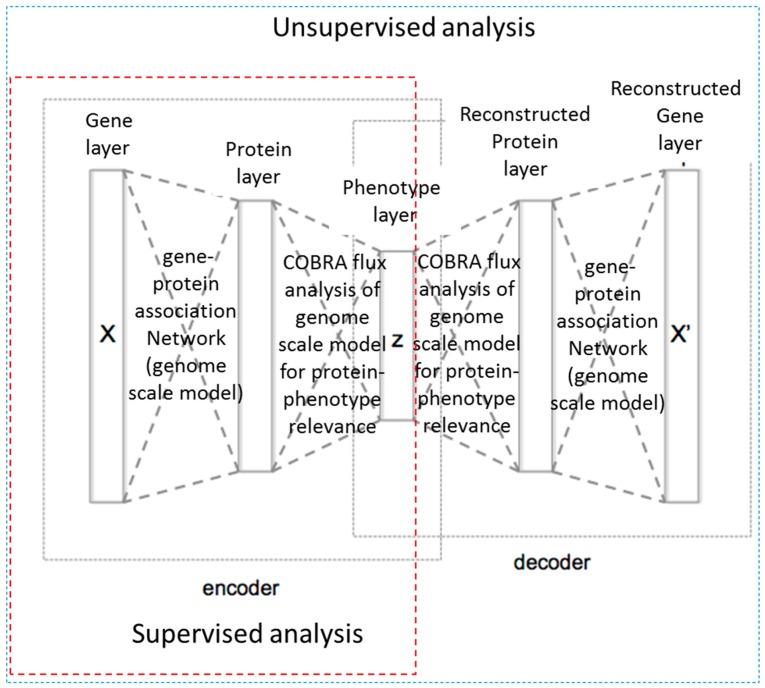
Schematic representation of the DeepMetabolism approach by Guo et al. [81].

In order to try and take advantage of the large amount of sequencing data and detailed genome scale models for more accurate phenotype prediction, Guo et al. [81] have developed and made available a biology-guided deep learning system: DeepMetabolism. DeepMetabolism integrates unsupervised pre-training and supervised training and, based on the test presented by the authors, provides a method for high accuracy, high speed and high robustness determination of phenotypes from genotypes. In this approach, the authors use biological knowledge to guide the design of the neural network structure. The authors build an autoencoder model with five layers from transcriptomics data where the first three layers belonged to the encoder part, modelling connections from gene expression to phenotype, and the last three layers belonged to the decoder part, which models the connection from phenotype to gene expression. The first layer represents the expression level of essential genes for *E. coli* metabolism; the second layer represents the abundance of resulting essential proteins; third layer represents the phenotypes of *E. coli*; the fourth and fifth layers were reconstructed protein and gene layers, respectively. Layers in the presented work were not fully connected. Instead, the authors used biological knowledge to define rational connections, thereby reducing the risk of over-parametrization. Connections between the first, second, as well as fourth and fifth layers were built from gene-protein associations from the genome-scale metabolic model of *E. coli*; connections between the second and third and the third and fourth layers was determined from COBRA analysis of the genome-scale model of *E. coli* used to identify proteins that were essential for given phenotypes. A schematic presentation of the deep network created is shown in Figure 6.

Machine learning methods have also been utilized for pathway prediction, showing good performance when compared with standard methods presented in the hard-coded pathway prediction tool PathoLogic, while at the same time allowing easier extensibility, tenability and explanation of the results [82]. Machine learning algorithms tested in the pathway prediction analysis included naive Bayes, decision trees and logistic regression. The goal of this study was to test methods for the determination of the presence of metabolic pathways based on the pathway information for many organisms presented in the pathway collection MetaCyc and to develop predictors for the presence of metabolic pathways in newly-sequenced organisms. In the course of this work, the authors have developed the gold standard pathway prediction dataset that was used for method validation and is made available for further pathway prediction work. Analysis of the performance of machine learning methods for pathway prediction have also shown that a small number of features contains the most information about the occurrence of pathways in an organism, with the most informative numeric value being the fraction of reactions along the path from an input to an output compound.

## 4. Conclusions

Experimental analyses measure values, i.e., concentrations, of variables in a model, e.g., metabolite concentrations or gene or protein expressions. In order to define the model, it is necessary to obtain information about the parameters that control the changes in these variables. Solving the system identification problem, i.e., determining parameters from variables, is often very strongly underdetermined with many parameters that need to be resolved from few variables in addition to many possible combinations of parameters, leading to the obtained values for variables. Mechanistic models use expert knowledge to derive information and develop models from targeted experiments. However, the large amount of data makes the application of data-driven, machine learning methods a highly attractive option. In addition, metabolic models can provide a large amount of data, and deriving actionable information from these model results can also greatly benefit from machine learning approaches. Thus, recent developments in machine learning and metabolism modeling compounded by the huge increase in computer power accessible for this task have made it possible to link data-driven and mechanistic models. 

Machine learning algorithms are able to learn optimal solutions for the analysis of new data from previously determined information. Without a doubt, with the availability of more advanced algorithms and the mounting availability of data, machine learning will be increasingly relevant for the analysis of metabolism. One of the major applications of metabolism models is in metabolic engineering where models can test genetic and regulatory changes towards increased productivity and reduced cost of production. Metabolic network models have an important role in helping determine targets, the side effect and toxicities of either drugs or toxins, through their direct effects or side-effects, as well as test possible toxic effects of chemicals in the environment. Machine learning can be implemented to integrate metabolic networks with available data. A recent example presented by [83] shows an application of machine learning and genome-scale models for the determination of drug side effects using the data available for human diseases, drugs and their associated phenotypes in order to determine metabolically-associated side effect predictors. Relational and logic-based machine learning was successfully utilized as part of the MetaLog project to provide causal explanations of rat liver cell responses to toxins using high throughput NMR metabolomics data collected as part of the Consortium for Metabonomics Toxicology (COMET) [79,84,85]. Passive machine learning methods compare offline, previously obtained information or data with models. With a highly dynamic system, with changing gene expression levels and different inhibitory or activating processes, it will be necessary to perform active learning that can generate new perturbations in a continual process. One of the first examples of an automatic generation of symbolic equations for a nonlinear coupled dynamical system directly from time series data was presented by Bongard and Lipson [86], where, for the first time, the authors have presented a method that models each variable separately. Symbolic modeling was shown to have explanatory value, suggesting that this automated reverse engineering approach leading to model-free symbolic nonlinear system identification may provide important help in understanding increasingly complex systems. In this example, model learning and optimization was combined with continual input of sensor data. This type of approach will be increasingly of interest with a growing availability of sensor information. 

Metabolism modeling can also be utilized for integrated analysis of different types of omics data, and some examples of the use have been shown in several recent reviews [87,88,89], outlining tools, problems, as well as applications in different fields, including medicine and biotechnology. 

In 2006, Kell [30] said: “By making mathematical models of the biological systems one is investigating (and seeing how they perform in silico) what is generally considered a minority sport, and one not to be indulged in by those who prefer (or who prefer their postdocs and students) to spend more time with their pipettes.” Over the last decade, there has been a major growth in the understanding of the value that computational biology models can bring to life sciences. Combining metabolomics with data-driven machine learning has a great potential in assessing the current or near future state of biological systems, but also, when combined with modelling methods, to predict future risks and events. There is no better time than the present to pick up this “minority sport”. 

## Figures and Tables

**Table 1:** Groups of machine learning algorithms based on algorithm similarities. Groups are based on [4,5]. Sample examples of the metabolomics application of methods are provided in the references included.

Algorithm Group	Short Description	Methods and Some Metabolomics Uses
Regression algorithms [6,7]	Iteratively improve the model of the relationship between features and labels using the error measure	Ordinary Least Squares Regression (OLSR); linear regression; stepwise regression; Local Estimate Scatterplot Smoothing (LOESS)
Instance-based algorithms [8,9]	Compare new problem instances (e.g., samples) with examples seen in training.	k-Nearest Neighbors (kNN); Self-Organized Map (SOM) and Locally Weighted Learning (LWL); SVM
Regularization algorithms [10,11]	An extension to other models that penalize models based on their complexity generally favouring simpler models.	Least Absolute Shrinkage and Selection Operator (LASSO) and elastic net
Decision tree algorithms [6,12]	Trained on the data for classification and regression problems providing a flowchart-like structure model where nodes denote tests on an attribute with each branch representing the outcome of a test and each leaf node holding a class label.	Classification and regression tree (CART); C4.5 and C5.0; decision stump; regression tree
Bayesian algorithms [13]	Application of Bayes’ theorem for the probability of classification and regression.	Naive Bayes, Gaussian naive Bayes, Bayesian Belief Network (BBN); Bayesian Network (BN)
Association rule learning algorithms [14]	Methods aiming to extract rules that best explain the relationships between variables.	A priori algorithm; Eclat algorithm
Artificial neural network algorithms including deep learning [15,16]	Building of a neural network.	Perceptron back-propagation Hopfield network Radial Basis Function Network (RBFN) Deep Boltzmann Machine (DBM) Deep Belief Networks (DBN) Convolutional Neural Network (CNN) stacked auto-encoders
Dimensionality reduction algorithms [17,18]	Unsupervised and supervised methods seeking and exploiting inherent structures in the data in order to simplify data for easier visualization or selection of major characteristics.	Principal Component Analysis (PCA) Principal Component Regression (PCR) Partial Least Squares Regression (PLSR) Sammon mapping Multidimensional Scaling (MDS) projection pursuit Linear Discriminant Analysis (LDA) Mixture Discriminant Analysis (MDA) Quadratic Discriminant Analysis (QDA) Flexible Discriminant Analysis (FDA)
Ensemble algorithms [19]	Models composed of multiple weaker models that are independently trained leading to predictions that are combined in some way to provide greatly improved overall prediction.	boosting bootstrapped aggregation (bagging) AdaBoost stacked generalization (blending) Gradient Boosting Machines (GBM) Gradient Boosted Regression Trees (GBRT) random forest

**Table 2:** Freely available software tools providing machine learning methods applicable to the metabolism analysis.

Tool Name	Focus	Availability
FingerID [21,42]	Molecular fingerprinting	http://www.sourceforge.net/p/fingerid
SIRIUS [43]	Molecular fingerprinting	https://bio.informatik.uni-jena.de/software/sirius/
Metaboanalyst [44]	General tool for metabolomics analysis	http://www.metaboanalyst.ca/
MeltDB 2.0 [45]	General tool for metabolomics analysis	-
KNIME *	General machine learning tool	https://www.knime.com/
Weka [46]	General machine learning tool	https://www.cs.waikato.ac.nz/ml/weka/
Orange *	General machine learning tool	https://orange.biolab.si/
TensorFlow	General machine learning tool	https://www.tensorflow.org/

* Visual programming languages made for easy software development without extensive programming knowledge.
